# Nematicidal activity of thermostable alkaline protease produced by *Saccharomonospora viridis* strain Hw G550

**DOI:** 10.1016/j.btre.2019.e00386

**Published:** 2019-10-25

**Authors:** Osama M. Darwesh, Ahmad S. El-Hawary, Usama S. El Kelany, Gamal M. El-Sherbiny

**Affiliations:** a Agricultural Microbiology Department, National Research Centre, Dokki, Cairo, Egypt; b Botany and Microbiology Department, Faculty of Science (Boys), Al-Azhar University, Nasr city, Cairo, Egypt; c Plant Pathology Department, Agricultural and Biological Division, National Research Centre, Cairo, Egypt

**Keywords:** *Meloidogyne incognita*, Thermostable alkaline protease, *Saccharomonospora viridis*, Nematicidal agent

## Abstract

Application of thermostable alkaline protease to control the harmful nematodes was investigated in the current study. A total of 14 proteolytic actinomycetes were isolated from Egyptian harsh environments. Out of them, isolate G550 exhibited the highest proteolytic activity (528.9 U/ml). Protease from isolate G550 exhibited high nematicidal activity against *M. incognita* under laboratory conditions and caused hydrolysis of J_2_S cuticle. This isolate was identified using molecular techniques and deposited in GenBank under name of *Saccharomonospora viridis* strain Hw G550 with accession number: MF152631. The G550 protease was extracted, characterized and applied under greenhouse conditions as nematicidal agent. This enzyme exhibited maximum activity and stability at alkaline pH (8) and thermal conditions (50–60 °C). Also, the results showed that, all treatments using protease caused a significant decrease in nematode reproduction and increasing in the plant properties. Finally, the thermo alkaliphilic protease could be used as bio-control agent against RKN.

## Highlights

• Isolation and identification of thermo alkaliphilic actinomycetes.
• Obtaining of thermostable alkaline protease enzyme.
• Evaluation of the nematicidal activity of obtained protease.
• Application of thermostable alkaline protease as nemticidal agent.

## 1 Introduction

Parasitic phytonematodes are virulent pests causing agronomic losses for many crops with approximately 14.6% of crop production [1]. There are more than 4100 spp. of phytonematodes have been reported as plant pathogens. Among them, root-knot nematodes (RKNs) are responsible for over than the half of damages caused by these parasites [2]. Due to their ability to infect almost all crops, RKNs as *Meloidogyne incognita* invasions resulted in losses equal to $400 million per year [3].

At present, management of RKNs is basically dependent on nematicides chemicals that have ecological hazards and are toxic to humans [4]. So, green alternative ways to control RKNs infections are necessary to be developed [5]. Modern technology based biology moves up to solve these problems by producing biotechnological agents as bioactive materials [[6], [7], [8], [9], [10]], nanobio-synthesized agents as silver, copper and zinc [11,12] and by producing non-traditional materials as antimicrobial agents [[13], [14], [15]]. One of these ways is biological control using microorganisms with potential active metabolites against RKNs. Many metabolites were recorded having activity against RKNs and produced by fungi [16], bacteria [17] and actinomycetes [18]. However, in contrast with fungi, bacterial metabolites with nematicidal activity are relatively rare and need more investigations in order to develop novel nematicidal metabolites [19]. In addition, it was reported that the nematicidal activity of microbial metabolites is related to their enzymatic potential especially chitinase and protease enzymes [20]. The nematicidal activity of these enzymes is attributed to the composition of nematode's cuticle or egg-shell that is mainly protein and chitin [21].

Nevertheless, the management of RKNs faces difficulties due to their nature as obligate root parasite that make most of their life cycle happened inside the host roots [22]. The life cycle of *M. incognita* is beginning with egg containing embryo that is developed to 1^st^ stage juvenile (J_1_S) which is hatched to the infectious 2^nd^ stage juvenile (J_2_S) in rhizosphere and then penetrate the host roots. The endophytic cycle starts after entrance of J_2_S and then developed to J_3_S and J_4_S then finally to the adult stage [23]. Accordingly, J_2_S is the most suitable phase for controlling. For that, J_2_S of RKNs is targeted for controlling as it lives for a short time outside the root.

Therefore, this study was focused on the ability of thermostable alkaline proteases produced by different actinomycetes that isolated from Egyptian habitats to kill *M. incognita*. In addition to isolation, purification and characterization of protease enzyme that showed the highest nematicidal activity against RKNs.

## 2 Materials and methods

### 2.1 Meloidogyne incognita (Root-knot nematode)

The pure culture of *Meloidogyne incognita* was obtained from phytonematodes research Lab., plant pathology department, National Research Centre, Egypt. A single egg mass picked from infested tomato seedling, *Lycopersicon esculentum* (Super strain B) was cultivated in plastic pots (15 cm, diam.) filled with autoclaved loamy-sand: clay (1:1 w/w). After 45 days of incubation and the appearance of disease symptoms, soil and root of the infected seedling were used as inoculants for new seedlings. Pots were kept under greenhouse conditions. They were watered as needed by tap water and a nutrient solution weekly. This served as mother culture and a culture bulk multiplication of the nematode. Identification of root-knot nematode females from the pure culture was confirmed based on perineal pattern morphology according to Netscher and Taylor [24]. The identification was done under light microscope according to Eisenback et al., [25]. For isolation of *M. incognita* second stage juveniles J_2_S (the infective stage), soil of the infected seedling was soaked in distilled water with good mixing followed by settlement for 5 min, then the water phase passed through a 200-mesh sieve and descended over a 325-mesh sieve to collect J_2_S.

### 2.2 Thermostable alkaline proteases actinomycetes producers

A total of 106 soil samples from harsh environments in Egypt were collected. The collected samples were sieved to remove various contaminant materials. Then the samples were air-dried and mixed with CaCO_3_ (1 g/100 g soil) for 24 h at 28 °C before plating to increase the numbers of actinomycetes [26]. Isolation of the thermostable alkaline proteases producing actinomycetes were performed by dilution plate technique using basal mineral salts agar medium supplemented with 1% casein [27]. Diluted samples were inoculated under aseptic conditions and then incubated for 7 to 14 days at 55 °C with pH 8.5. Selected colonies (rough, chalky) of actinomycetes were transferred from mixed culture onto respective agar plates and incubated for another 7 days. Plates containing pure cultures were stored until further examinations.

### 2.3 Detection and production of protease enzyme

All isolates were cultivated overnight in 250 ml flask containing 125 ml nutrient broth medium with a final pH of 8.5, at 55 °C under shaking of 200 rpm. Ten milliliters from the overnight cultures were re-cultivated in 250 ml flask containing 125 ml complex medium (Glucose, 1%; Yeast extract, 0.5%; Peptone, 0.25%; Casein, 0.25%; MgSO_4_, 0.03%; FeSO_4_, 0.002%; ZnSO_4_, 0.02%; CaCO_3_, 0.1%; KH_2_PO_4_, 0.1%; K_2_HPO_4_, 0.1) and incubated at 55 °C for 96 h at 200 rpm shaking conditions [28]. By the end of incubation, cells were separated by centrifugation (1789 xg, 10 min, 4 °C) and the enzymes cell-free extracts were collected. After removing the cells biomass, 4:1 v/v of cold acetone was added drop-wise to supernatants under continuous stirring in presence of ice bath. The solutions were left in refrigerator overnight to enable the protein precipitation. The precipitated crude enzyme was separated by centrifugation under cooling at 4025 xg for 10 min. The precipitated enzyme was dissolved in potassium phosphate buffer (pH 8.5) and stored for further studies [29].

### 2.4 Enzyme assay

Protease activity of the proteolytic isolates was determined according to Folin and Ciocalteu [30] using L-tyrosine as a standard. Five milliliters of 0.65% (w/v) casein in 50 mM potassium phosphate buffer, pH 8.5 was added to 1 ml enzyme solution and the mixture was incubated for 10 min at 55 °C in the water bath. After incubation, 5 ml of 110 mM trichloroacetic acid (TCA) reagent was added to enzyme-substrate solution to terminate the reaction. The mixture was put in ice bath for 10 min and then centrifuged at 13,500 rpm, at room temperature for 10 min then the supernatant was collected. The color development reaction was done by adding 2 ml of supernatant to 5 ml of 500 mM sodium carbonate solution followed by addition of 1 ml of Folin Ciocalteu’s phenol reagent into a tube and mixed by swirling. The reference tube had the same reaction except the enzyme solution. The activity was recorded using spectrophotometer by changing in absorbance at 660 nm. The enzyme activity unit (U) was calculated by one unit is that hydrolyze casein to produce color equivalent to 1.0 μmole (181.0 μg) of tyrosine per min under the defined assay conditions [30,31].

### 2.5 Meloidogyne incognita mortality assay

The protease crud enzyme extracts of the proteolytic isolates were evaluated for their nematicidal activity against *M. incognita* J_2_S under laboratory conditions by mortality test at different times of exposure (24, 48 and 72 h). For mortality test, 4 ml sterile distilled water containing 100 ± 5 freshly hatched J_2_S of *M. incognita* were placed into 15 ml sterile screw-capped tube containing 100 μl from each crud enzyme extract diluted by 900 μl potassium phosphate buffer (pH 8.5). In addition, control groups were contained the second stage larvae in distilled water plus boiled crud enzyme [32]. Each extract and its check control were replicated five times. All tubes were kept in incubator at 30 °C. Numbers of survived and dead larvae were counted every 24 h using one ml counting slide. Nematodes were considered alive if they moved or assumed a winding shape and it is dead if they were straight and immobile. To assess mortality after 72 h of exposure, the nematodes in all treatments were washed on a 20 μm aperture sieve and transferred to clean tubes and then incubated for an additional 24 h to see whether immobile nematodes resumed activity or not [2]. The collected data were calculated to determine the percentage of mortality for each extract using equation of mortality %= [(The number of live nematodes larvae in the control) – (The number of live nematodes larvae counted in the treatments)/ (The number of live nematodes larvae in the control] ×100.

### 2.6 Greenhouse experiments

Protease produced by isolate G550 was selected based on its nematicidal activity in the *in vitro* experiment for further evaluation under greenhouse conditions. Plastic pots of 15 cm diameter were filled with 1 kg sterilized mixed soil from loamy-sand: clay (1:1 w/w). One-month old eggplant seedlings were transplanted into the center of each pot as one seedling/pot. The selected crude enzyme extract was applied to soil as one-time drench with 10 ml/ pot and 2000 freshly hatched second stage juveniles of *M. incognita* were added in three holes around the root. The treatment was done using three times of nematode infections (enzyme and nematodes at once, enzyme treatment a week after and enzyme treatment before infection). Each treatment was replicated 5 times. The experiment was set on greenhouse bench at 30 ± 2 °C. Plants were watered slightly after inoculation and thereafter, whenever required. The experiment was terminated 60 days after nematode inoculation. Numbers of galls, egg masses, females and developmental stages as well as number of eggs/egg mass were counted, percentages of reduction were calculated. Eggs/egg mass were recorded by detaching 10 egg masses from infected roots using needle and exposed to sodium hypochlorite (2%) for 3 min and released eggs were counted to determine number of eggs /egg mass. The number of females and developmental stages /root was estimated by investigation under stereo microscope after scratching of roots with needle. After stereoscopic examination, roots were cut into small pieces and homogenized in a homogenizer at adequate speed for 40 s and the released nematodes were counted under light microscope. For J_2_S detection, soils of each treatment were carefully mixed to compose typical samples of 250 g. Nematodes were extracted using sieving and decanting technique. Extracted J_2_S were counted in 1 ml suspension using counting slide under light microscope and repeated four times, then the mean was calculated. Eggplant growth parameters were recorded as root and shoot lengths, fresh and dry shoot weights.

### 2.7 Characterization of enzyme activity and stability towards pH values

Studying of pH effect on the G550 protease activity was investigated using different buffer systems at 55 ºC. The optimum pH of the tested enzyme was determined under the standard assay conditions by measuring activity in the presence of buffers at different pH values (4, 5, 6, 7, 8, 9, 10, 11). For pH stability, the enzyme was incubated in the previous pH buffers for 24 h at room temperature. The relative activity was determined before and after incubation. The percentage of remaining activity was calculated [28]. Buffers used for this purpose were Glycine-HCl buffer (pH: 4 and 5); Citrate–Phosphate buffer (pH: 6); Phosphate buffer (pH: 7 and 8); Glycine–Sodium hydroxide buffer (pH: 9 and 10); Sodium bicarbonate–Sodium hydroxide buffer (pH: 11).

### 2.8 Characterization of enzyme activity and stability towards temperature

The effect of temperature on the enzyme activity was investigated using standard assay conditions at pH 8 (phosphate buffer). The optimum temperature of the tested enzyme was determined by measuring the activity at different temperatures (30, 40 50, 60, 70 °C). For stability, the enzyme was incubated at different temperatures (30, 40 50, 60, 70°C) for 30 min. Enzyme activity was determined under the standard assay conditions and the percentage of remained activity was calculated [28].

### 2.9 Identification of the most active isolate against RKN

Isolate G550 exhibited the most nematicidal activity against *M. incognita* was identified based on morphological and biochemical characteristics and its 16S rRNA sequences. The results of morphological and biochemical identification of the selected isolate was confirmed through molecular and phylogenetic methods. The genomic DNA was extracted and the 16S rRNA gene was amplified by PCR using a Bio-Rad T100 thermal cycler (Bio-Rad Laboratories, CA, USA) as previously described [33,34]. The PCR products were purified using a QIAquick PCR purification Kit (Qiagen, USA). The purified 16S rRNA fragments were analyzed by agarose gel electrophoresis and visualized using UV-transilluminator [35,36]. Sequencing of the amplified 16S rRNA fragments were perform using a BigDyeR Terminator v3.1 Cycle Sequencing Kit (Applied Biosystems, Carlsbad, CA, USA) on an Applied Biosystems 3730xl DNA Analyzer. Similarities of the bacterial nucleotide sequences with other known sequences were examined by comparisons with the National Center for Biotechnology Information (NCBI) database for reference and type strains using the BLASTN program (https://blast.ncbi.nlm.nih.gov/Blast.cgi). A phylogenetic tree based on partial 16S rRNA sequences was constructed using the neighbor-joining method contained within the Clustal X program and MEGA6 software. The obtained sequences were submitted to GenBank [37].

### 2.10 Statistical analysis

The data was collected from three or five replicates based on the method and submitted to analysis of variance (ANOVA) and compared by the Tukey test with significance level of 5%. MINITAB statistical software version 18.1 (Minitab, Inc., PA, USA) was used for the analysis [36].

## 3 Results and discussion

### 3.1 Isolation of extreme-protease producing actinomycetes

**Fig. 1:**
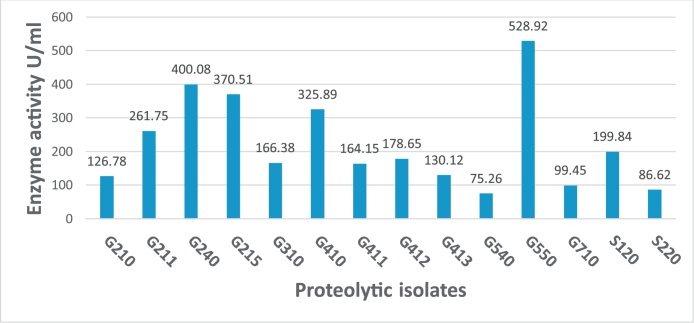
The protease activities of crud enzyme extracted from proteolytic isolates.

One of the most essential steps for isolation of the extremozymes producers is the isolation samples collection. First of all, this study was concerned with isolation of extreme-protease producing actinomycetes and investigation of their activity against parasitic nematodes. According to this goal, the samples sites were chosen to be extremely harsh environment with different ecological characteristics to increase the diversity of microorganisms. Egypt has many harsh environments that might be a good source for the extremophiles [38]. A total of 14 proteolytic actinomycetes were isolated. These isolates were selected according to the variation of distinct colony characteristics like size, pigmentation, opacity, texture, form, elevation and margin surface on culture agar medium. The obtained isolates were determined their ability to produce proteases enzymes. The standard curve of L-tyrosine to determine protease activity was done. The optical densities (OD) of colors resulted from ten different concentrations of L-tyrosine was plotted and produced trend line equation with high determination coefficient (R^2^) value of 0.9523 that used for determination of proteolytic activity of each isolate. After calculation of crud enzymes activity, the results showed that isolate G550 exhibited the highest proteolytic activity against casein with 528.9 U/ml. However, there are 4 isolates showed high proteolytic potentiality above 300 U/ml. The lowest protease activity was observed by isolate G540 with 75.2 U/ml **(**Fig. 1**)**. The results of protease activity assay of obtained isolates were compatible with data demonstrated by Sharma et al. [39].

### 3.2 Production of protease from the proteolytic actinomycetes

Thermo-alkali protease enzyme was produced by proteolytic actinomycetes that isolated from harsh environments in Egypt. After removing of bacterial cells by centrifugation, the supernatant was used as a source of enzymes. The crude enzymes, mainly proteases, were extracted using chilled acetone. The crude enzymes were re-suspended in the suitable amounts of phosphate buffer and then applied as nematicidal agent. Global researchers are going forward toward applying the safe and environmentally technologies for controlling crop pathogens. This called smart agriculture like bioactive agents [13,40] and nanobiotechnology [9,41].

### 3.3 Nematicidal activity of proteases against *M. incognita* J2S at *in vitro* level

**Fig. 2:**
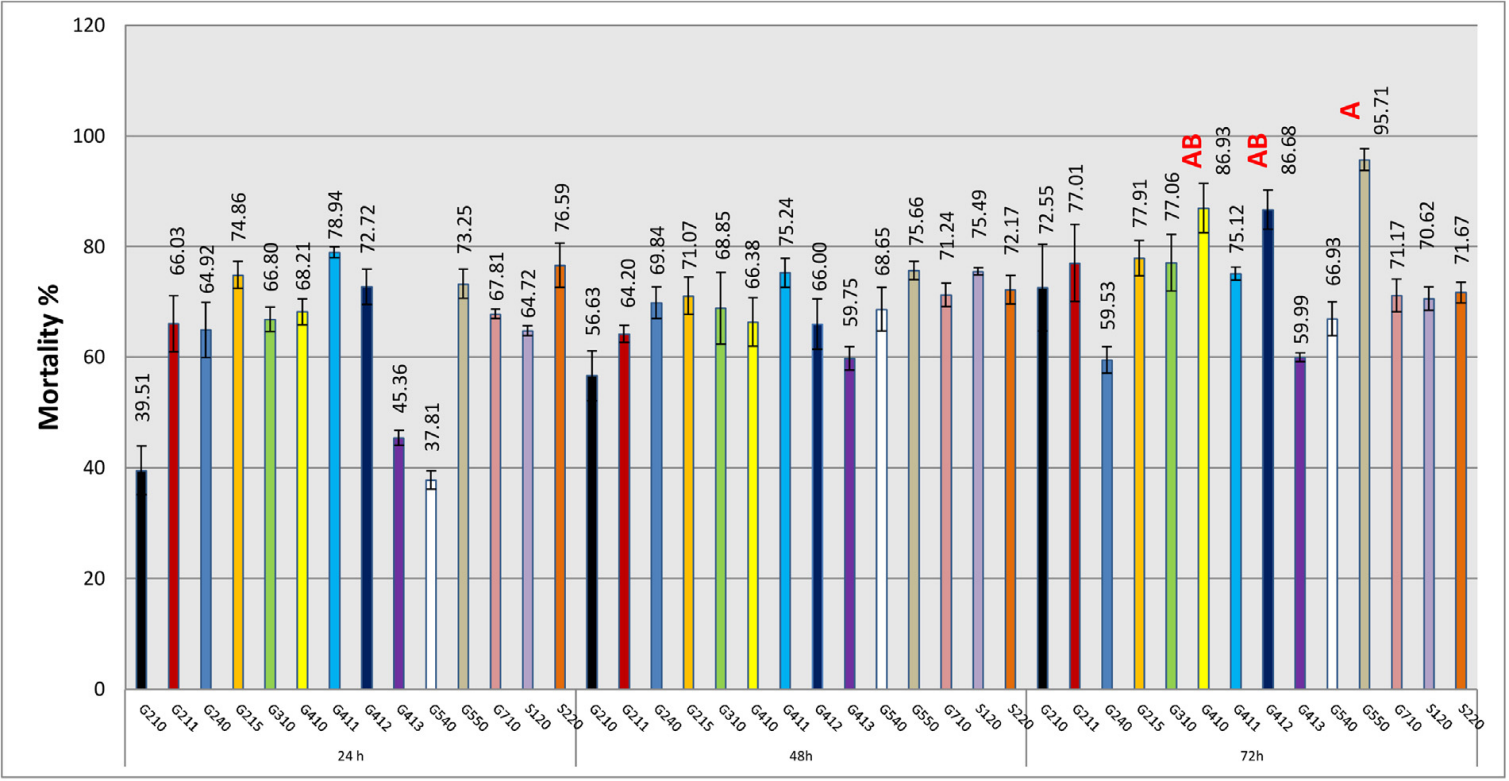
The percentage of mortality caused by protease enzymes of the proteolytic isolates after 24, 48 and 72 h and the highest significant activity resulted from Tukey test (p ≤ 0.05) was labeled with letter 'A'.

**Fig. 3:**
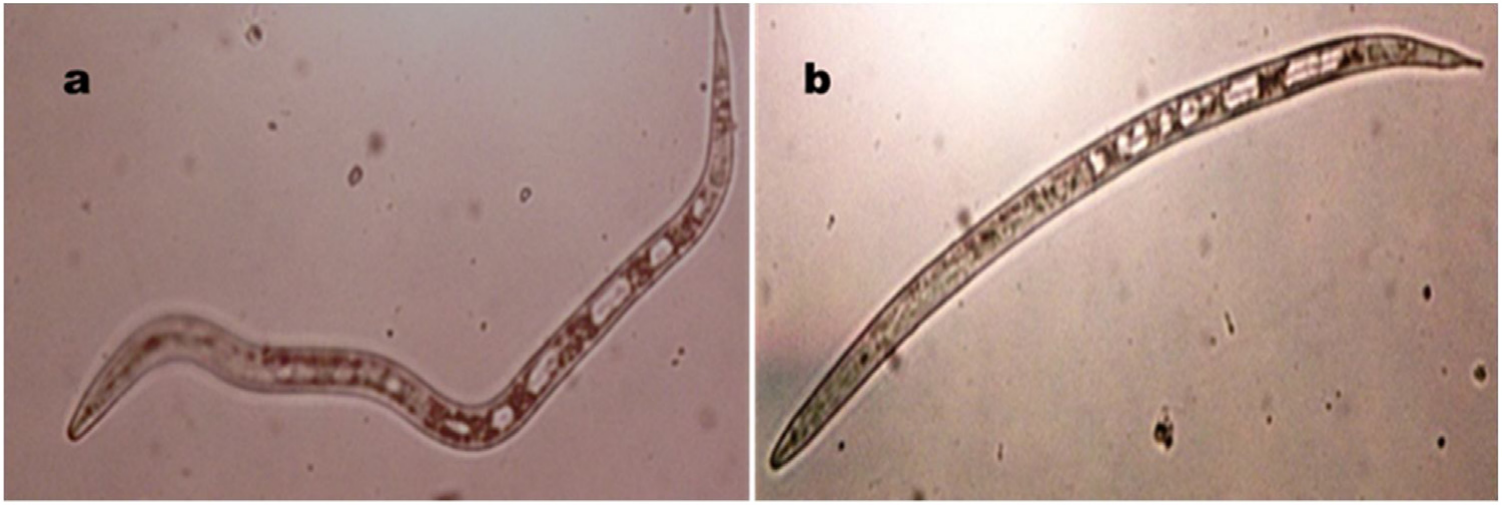
a; Motility (winding shape) of J2S indicate the viability observed under light microscope (200X), b; Absent of movement (straight shape) indicate the mortality of J2S (200X).

**Fig. 4:**
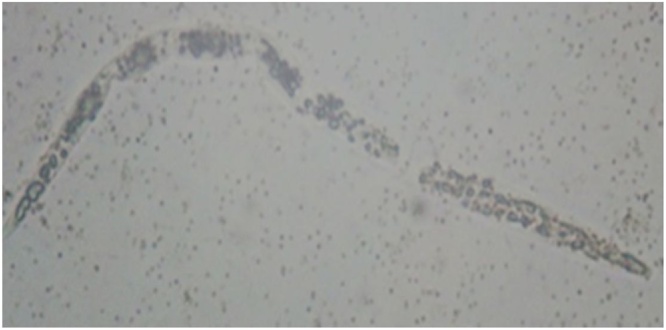
Destruction and rupture of J2S intestine and cuticle caused by proteolytic effects of isolate G550 after 48 h of treatment (200X).

The main goal of the current study is to obtain bioactive protease against the harmful plant parasite which decrease the crop productivity (*M. incognita* J_2_S). The obtained enzymes were applied as bio-pesticide to kill nematodes. Based on the bioassay results, all extracts under investigation showed nematicidal effects against *M. incognita* J_2_S with variation in their potentialities to kill. In general, the increase in the juveniles' mortality positively correlated with the increasing of exposure period with few exceptions. The mortality percentage of nematode larvae ranged from 37.81 to 95.7%. Amongst 14 protease extracts, the maximum mortality percentage was observed by isolate G550 with 95.7% after 72 h and it was lettered by 'A' as it was the highest significant value. Also, proteases produced by isolates G410 and G412 exhibited activity next to G550 with 86.9 and 86.6%, respectively and were at the same significant levels **(**Fig. 2**)**. The proteolytic activity of the extracts under study exhibited different nematicidal effects on J_2_S. As shown in Fig. 3, the difference between live and dead J_2_S is obvious by observation of J_2_S movement. In addition, the hydrolysis of J_2_S cuticle and internal digestive system were remarkable for many proteolytic extracts especially from the second day of the treatment **(**Fig. 4**)**.

### 3.4 Identification and phylogenetic analysis of isolate G550 as the best producer of protease

**Fig. 5:**
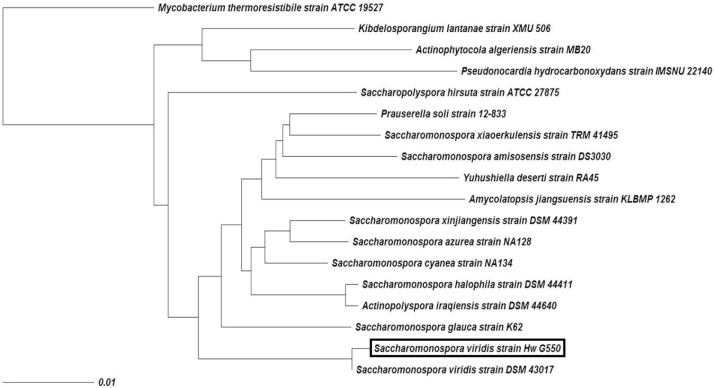
Neighbour-joining phylogenetic tree, based on 16S rRNA gene sequences of *Saccharomonospora viridis* strain Hw G550.

Previously published morphological and biochemical characteristics about isolate G550 and showed that it is similar to *Saccharomonospora viridis* [42]. In this study, the identification was confirmed by 16S rRNA sequencing. After amplification and sequencing of 16S rRNA gene, blast program (https://blast.ncbi.nlm.nih.gov/Blast.cgi) was applied to assess the DNA similarities. The search in NCBI results this isolate, G550, showed high similarity to *Saccharomonospora viridis* strain DSM 43,017 with 94% identity. Subsequently the partial sequence of this strain was deposited in Genbank as *Saccharomonospora viridis* strain Hw G550 16S ribosomal RNA gene, with accession number of MF152631. A phylogenetic tree depending on the alignments of the isolate was displayed using TREEVIEW program and represented in Fig. 5. The data reported in Bergey’s manual systematic bacteriology second edition volume five, the actinobacteria, part A, by Goodfellow et al. [43] for description of family *Pseudonocardiaceae,* showed complete identity with the data given through isolate G550. In addition, the data reported by Nonomura and Ohara [44] for description of *Saccharomonospora viridis* showed great similarity with the results exhibited by isolate G550 as it was characterized by the dichotomously branched mycelium, and sporophores with single pine-cone spores [45].

### 3.5 Characterization of G550 protease

**Fig. 6:**
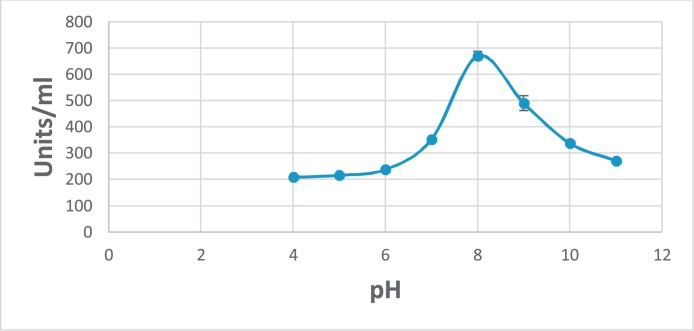
Effect of pH on G550 protease activity.

**Fig. 7:**
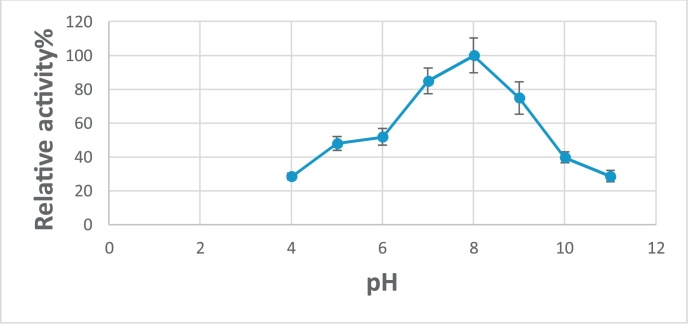
Effect of pH on G550 protease stability.

The important concern now in the global scientific area is to obtain the stable microbial agents against harsh conditions like pH and temperature. Egypt soil is considered a thermo and alkaline, for that we should buffering this soil when using microbial enzymes or choosing thermostable alkaline enzymes [29,46]. This may be investigated in this study. In case of alkaline stability, the highest activity of G550 protease against casein was exhibited at pH 8.0 (Fig. 6). The protease enzyme was stable over a range of pH between 7.0 and 9.0 and retained approximately 50% of its original activity after incubation in buffer with pH 5.0 and 40% at pH 10.0 (Fig. 7).

**Fig. 8:**
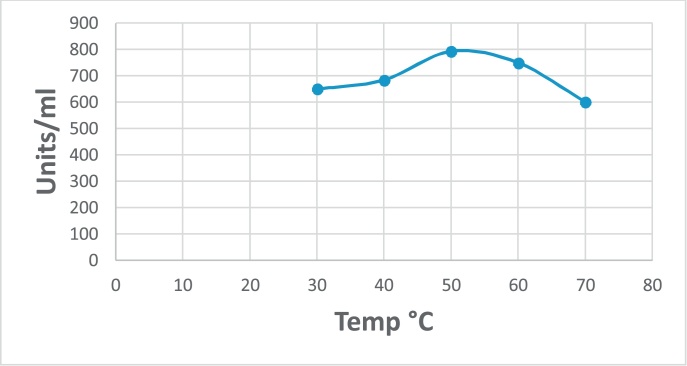
Effect of temperature on G550 protease activity showing the optimum temperature is 50 °C.

**Fig. 9:**
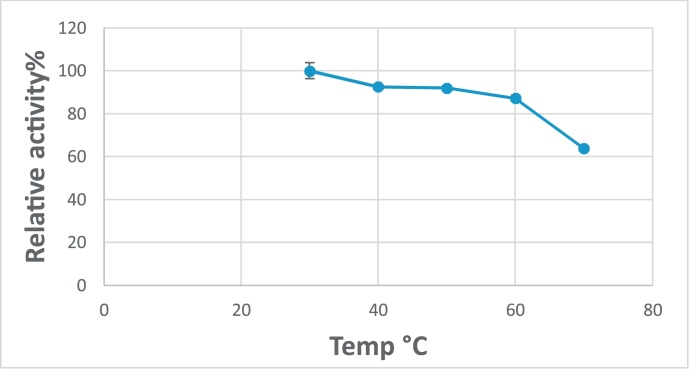
Effect of temperature on G550 protease stability.

Also, the effect of temperature on G550 protease activity was studied and the results showed that the highest activity was at 50 ºC (Fig. 8). The G550 protease enzyme was also stable with about 90% of its activity after thermal treatment at 40 and 50 °C for 30 min (Fig. 9). Reduction in activity was clearly observed after incubation at 70 °C with 40% reduction. From the previous results, we can conclude that the G550 protease is thermostable alkaline enzyme and it is suitable for applying in Egyptian soils.

Temperature and pH play important roles in enzyme activity and stability. The effects of pH and temperature on the activity and stability of most potent protease were investigated. The enzyme exhibited maximum activity at alkaline pH indicating that the enzyme was active in alkaline environment. For stability, G550 protease exhibited stability at wide range of temperature (30–70 °C). The enzyme in this study was more suitable in alkaline media than the enzymes from *Bacillus amyloliquefaciens* V656 [47]. Also, most of enzymes produced by actinomycetes showed high activity in alkaline conditions like enzymes of *Streptomyces* sp. CS495 that were active from pH 8.0 to 13.6 with the highest activity at pH 12.5 [48], but the tested enzyme is stable at thermal conditions plus alkaline one. These results are compatible with data revealed that the enzymes worked under alkaline conditions with high potentiality and could be used in many applications [49,50].

### 3.6 Nematicidal activity of G550 protease against RKN and improving of eggplant properties

**Fig. 10:**
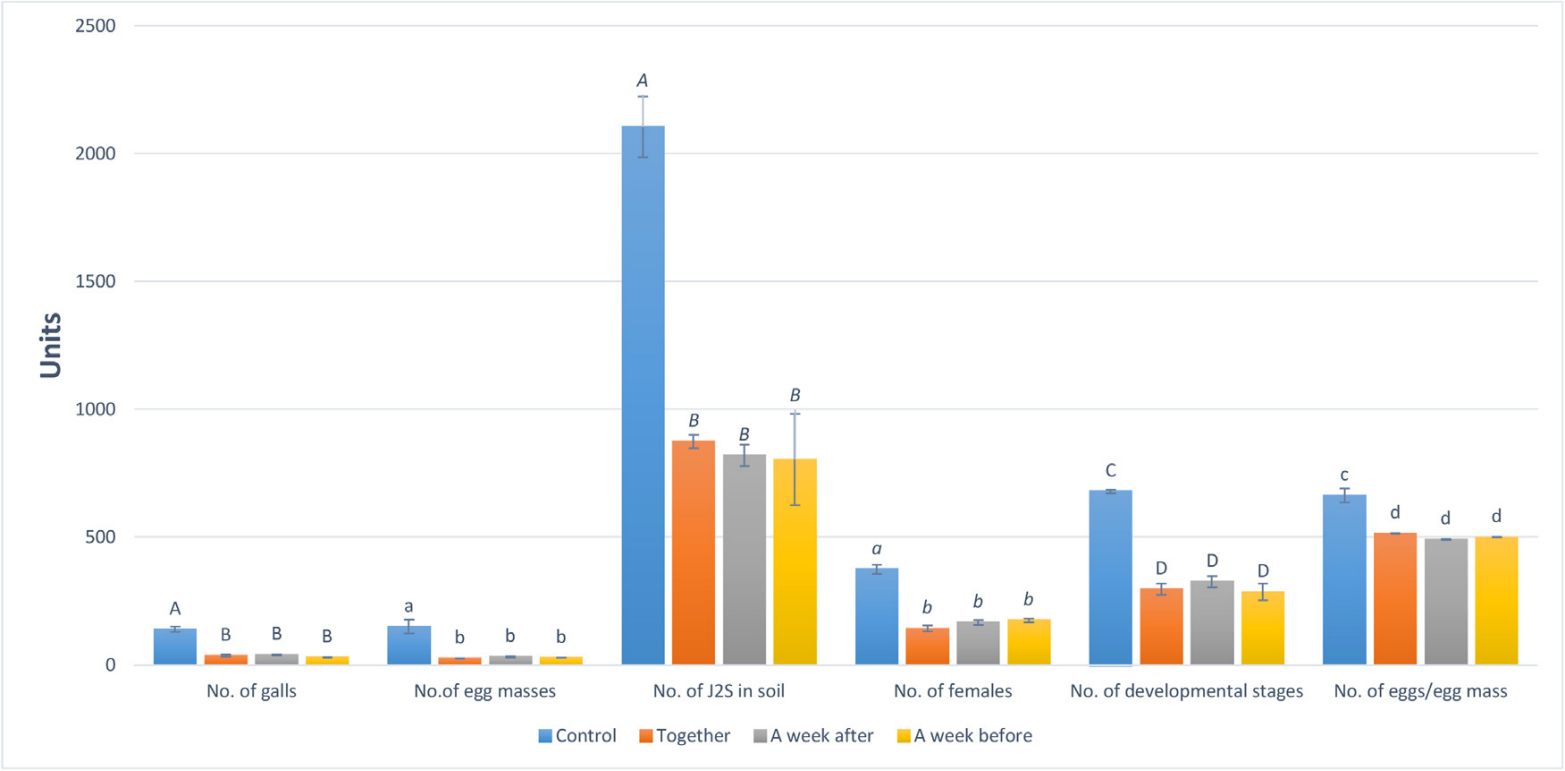
Effect of isolate G550 protease as soil drench treatment on *in vivo* reproduction of M. incognita in eggplant (Values not sharing letters are significantly different).

**Fig. 11:**
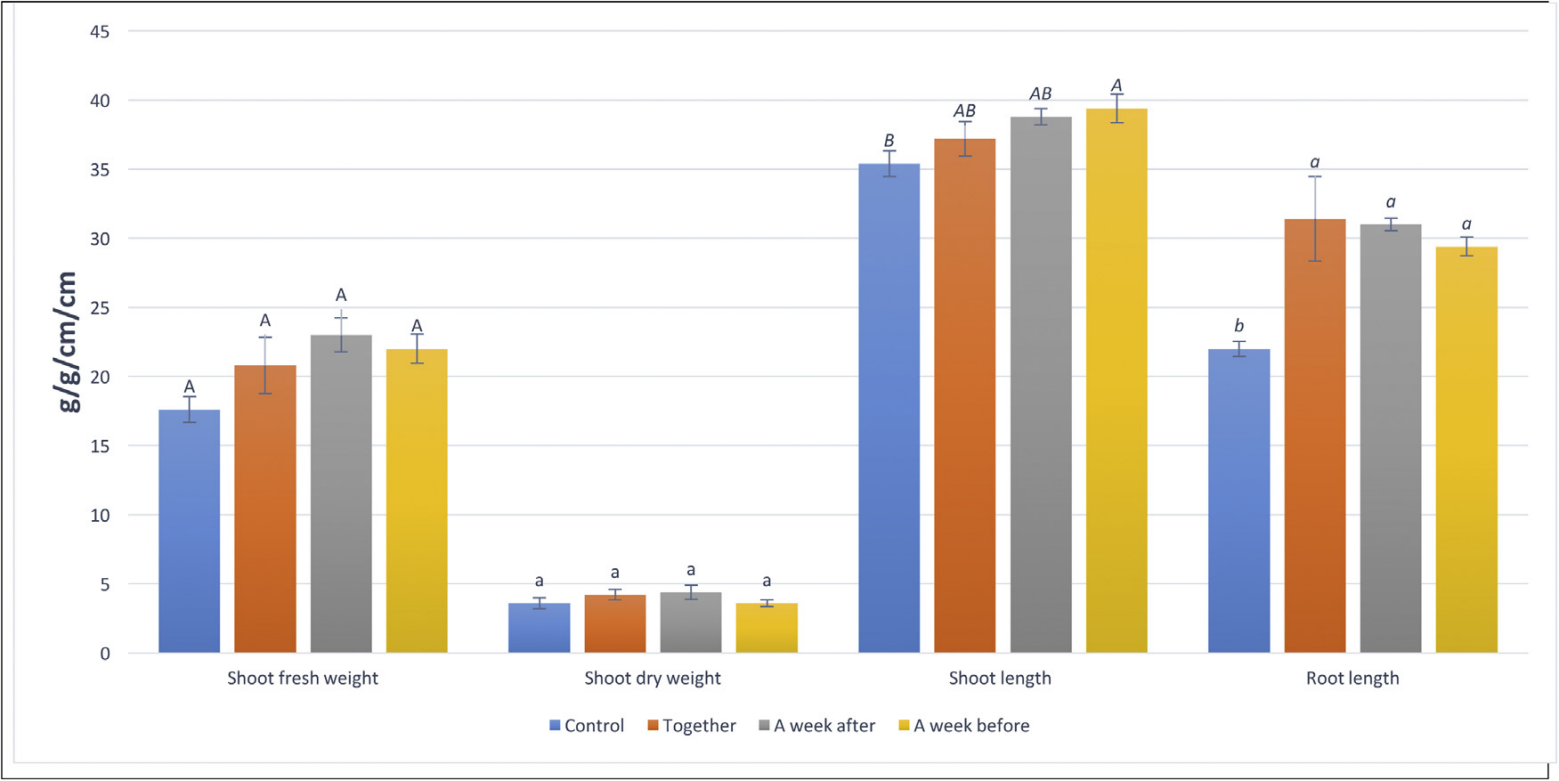
Effect of isolate G550 protease as soil drench treatment on growth parameters of eggplant infected with M. incognita (Values not sharing letters are significantly different).

Under greenhouse conditions, the selected hydrolytic enzyme induced a significant reduction in *M. incognita* reproduction on eggplant as soil drenching. The results showed that, all treatments caused a significant (p ≤ 0.05) decrease in nematode reproduction as indicated by the reduction percentage in number of females, egg-masses, galls and developmental stages /root as well as number of larvae /pot as compared to untreated control (Fig. 10). These findings confirmed the results of Zavaleta-Meija and Van Gundy [51] and Becker et al. [52], who found that, when tomato, cucumber and clover treated with rhizobacteria as soil drench treatments they suppressed significantly in penetration of nematodes inter the roots and reduced the root galling of *M. incognita* under greenhouse conditions. All treatments with G550 protease caused significant increasing in root length. In general, all values of growth parameters were higher than those of control if significance was neglected with one exception (shoot dry weight of protease treatment week before infection). In addition, treatment with protease significantly increased shoot length when applied a week before of nematode infection. Also, the treatment by protease caused maximum increasing in plant shoot fresh and dry weights in a treatment of week after of nematode infection with 30.86 and 22.22%, respectively, while the maximum increasing in plant shoot and root length were caused by protease treatment a week before and together with nematode infection with 11.3 and 42.73%, respectively **(**Fig. 11**)**. Furthermore, the potentialities of *Bacillus* spp. in controlling plant pathogens were referred to their ability to produce cuticle-degrading proteases with nematicidal activity which play an important role in bacteria–nematode interactions and serve as important nematicidal factors in balancing nematode populations in the soil [[53], [54], [55]].

Finally, the *in vivo* study under greenhouse conditions confirmed the *in vitro* results although the treatment was as one-time soil drench. Therefore, these findings may be improved if used in many doses that are reported by El-Hamshary et al. [56]. His study revealed that using two doses from each enzymatic preparation gave best results in reducing nematode multiplication more than one dose. This suggests that, more than one dose from the enzymes are needed to maintain activity high enough to achieve long-term bio-control.

## 4 Conclusion

From the obtained data, the hydrolytic enzymes especially thermo alkaliphilic proteases produced in the current study could be used as bio-control agent against RKN. Enzymes resistant to extreme conditions showed great ability to inhibit and kill harmful parasite under study in a promising manner, which qualifies them for further study to improve their performance in field conditions.

## Declaration of Competing Interest

Authors declare that there are no conflicts of interest.
